# Supplementary material for: An Indoor Gardening Planting Table Game Design to Improve the Cognitive Performance of the Elderly with Mild and Moderate Dementia
Source: Int J Environ Res Public Health. 2020 Feb 25;17(5):1483. doi: 10.3390/ijerph17051483 (PMC7084796; doi:10.3390/ijerph17051483)
Supplement: Supplementary file 1 [file ijerph-17-01483-s001.pdf]

1. Semi structured interview schedule:

Interview time:

October 29 ~ November 1, 2018 Monday ~ Thursday afternoon: 1400 ~ 1630

Interview location: Chaoyang care center, Douliu City

Interviewees: 14, 3 men and 11 women.

Time of each interview: 30 minutes

Interview planning:

1. Open interview for 10 minutes

Interview topic:

- Ask respondents to talk about the working background before retirement.
- Preference for gardening
- Interviewees talk about planting experience.
- Knowledge of seasonal flowers or plants in Taiwan.

Break for one minute

2. Mini-Mental State Examination for each participants for about 20 minutes.

3. Barthel Index scores were provided by the care center.

4. Interview time schedule:

Time<br>Date	14:00~1430	1440~1510	1520~1550	1600~1630
2018/10/29	P1<br>Mr. Chen<br>Jun-sheng	P2<br>Mr. Zhuang<br>Zhengtong	P3<br>Ms. Huang<br>Lin Azuan	P4<br>Ms .Shei<br>Chen Guihua
2018/10/30	P5<br>Ms. Xu<br>zhangjinzh	P6<br>Ms. Zhang<br>Yangrun	P7<br>Mr. Zhou<br>Kuncheng	P8<br>Ms.<br>Zheng<br>Sufang
2018/10/31	P9<br>Ms. Huang<br>Yuxue	P10<br>Ms.Lin<br>Chunju	P11<br>Ms. Chen Xi	P12<br>Ms. Liu Linsu
2018/11/1	P13<br>Ms. Huang<br>Shijiu	P14<br>Ms. Qiu<br>Chen Boji		

2. Experimental steps and details

- Low-level tasks: The equipment used in this experiment included a mobile phone for video recording, an indoor gardening game group, three objects for MMSE attention questions, and a behavior observation record of the elderly, as shown in Figure 2. There were three subtasks in the low-level stage. Before the formal experiment, participants were given a warm-up for three minutes. They were asked to recall the contents of the picture card presented by the experimenter. Then, the experimenter explained the step-by-step tasks for the gardening table games and the flower combination cards for the table games (Figure 2). After the experimenter explained the flower combinations and steps using the cards, and the actual operation was

shown to the participants, the flower combination task was carried out immediately. At this time, the participants were asked to speak out the flower parts and the combination process for selection to help focus their attention. In the low-level task, the picture card was placed on the desktop until the end of the task, and the flower parts were placed on the desktop in an unclassified form; then, the participants were asked to select the parts and complete them in combination. The first task consisted of 8 flowers, the second task increased to 14, and the third task increased to 20. In this step, the elderly patients' sustained attention was improved step by step, and we recorded the time from the beginning to the end, the correct rate of participant combinations, the number of prompts, and the time taken for continuous combinations.

- Medium-level task: the medium level task continued the primary level's flower combination task but increased the difficulty slightly. Five minutes before the formal start of the exercise, a warm-up task was performed, similar to the primary level stage of the "flower composition". In the medium level task, participants were required to watch the presented cards for 3 minutes, and the experimenter covered the cards. Then, the participants were required to select the same color flower parts presented by the cards and specify the matching colors of flowers, flower buds, and flower stems. This step was done to train both focus and selective attention. Like the primary level task, the first task consisted of 8 flowers, the second task increased to 14, and the third task increased to 20. The experimenter recorded the flower combination accuracy, the number of prompts, and the duration of the flower combination task.
- Higher-level task: The higher-level task was the same as the medium-level task, but with an indicator ring for color and digital tracking. The participants were asked to watch the picture card for 3 minutes after the 5-minute warm-up task of flower combinations. The participants were required, according to their memory, to search for the same color as the picture cards corresponding to the bottom color of the flower stem and corresponding to the digital color ring on the base, as shown in Figure 2. First, the participants used 8 flowers as basic training to improve their sustained attention in a gradual way. The subjects had to insert the base according to the specified color or number sequence and complete the task correctly. The second task increased to 14, and the third task increased to 20. The combination time, accuracy rate, number of prompts, and duration of participants in the task execution were recorded.

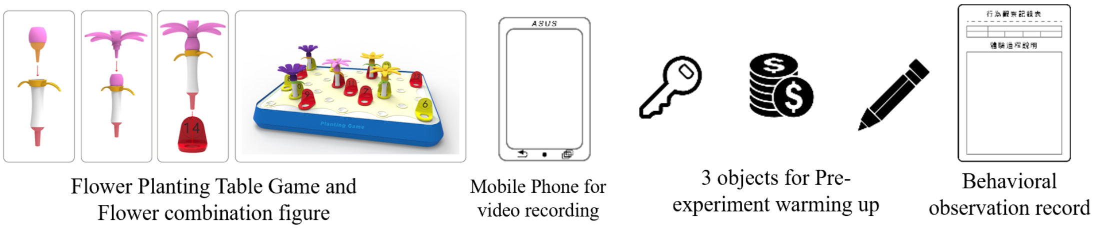

Figure 2. Equipment used in this research experiment.
